# The Performance, Ingestive Behavior, Nutrient Digestibility, Ruminal Fermentation Profile, Health Status, and Gene Expression of Does Fed a Phytochemical–*Lactobacilli* Blend in Late Pregnancy

**DOI:** 10.3390/ani15040598

**Published:** 2025-02-19

**Authors:** Amr A. Gabr, Fayek Farrag, Mohamed Ahmed, Yosra A. Soltan, Ahmed Ateya, Umar Mafindi

**Affiliations:** 1 Department of Animal Production, Faculty of Agriculture, Mansoura University, Mansoura 35516, Egypt; dr.amrgabr@mans.edu.eg (A.A.G.); farrag2000@mans.edu.eg (F.F.); umarmafindi2@gmail.com (U.M.); 2 Animal Production Research Institute, Agricultural Research Center, Ministry of Agriculture, Dokki, Giza 12619, Egypt; 3 Animal and Fish Production Department, Faculty of Agriculture, Alexandria University, Alexandria 21545, Egypt; 4 Department of Development of Animal Wealth, Faculty of Veterinary Medicine, Mansoura University, Mansoura 35516, Egypt; dr_ahmedismail@mans.edu.eg

**Keywords:** direct feed microbial, performance, health response, immune response, gene expression, does

## Abstract

This study investigated the effects of a phytochemical–*Lactobacilli* blend (PEL) on the performance and health of late-pregnant does. Thirty Zaraibi does (30.9 ± 0.37 kg body weight, 3–3.5 years old, 90 days pregnant) were randomly assigned to three experimental groups, ensuring equal distribution based on body weight, parity, litter size, and previous lactation milk production. The does were fed either a control diet (no supplementation) or a PEL-supplemented diet (control diet plus 2 g or 4 g of PEL per day) for 60 days. PEL supplementation linearly improved (*p* < 0.05) feed intake, nutrient digestibility, and ruminal concentrations of acetic and propionic acids, while significantly reducing (*p* < 0.05) ruminal ammonia-N concentrations. PEL supplementation linearly reduced (*p* < 0.05) feeding and rumination times while increasing idling time. Blood hematological parameters improved (*p* < 0.05) with PEL supplementation. Serum protein, liver and kidney function indicators, lipid metabolism, cortisol, and thyroid hormone levels were significantly improved (*p* < 0.01) in a linear manner with PEL supplementation. Antioxidant status, pro-inflammatory cytokines, and immune response were enhanced both linearly and quadratically (*p* < 0.05) with PEL treatment. Gene expression analysis revealed a linear upregulation (*p* < 0.05) of growth, immune, and antioxidant-related genes with PEL supplementation. These findings suggest that supplementing pregnant does with 4 g of PEL per day can effectively enhance their performance, health, and metabolic status.

## Simple Summary

This study investigated the effects of a phytochemical–*lactobacilli* blend (PEL) on the performance and health of late-pregnant does. Thirty pregnant does were assigned to three groups and fed either a control diet (no supplementation) or the control diet supplemented with 2 g or 4 g of PEL per day for 60 days. PEL supplementation enhanced feed intake, nutrient digestion, and overall health. Does receiving PEL spent less time eating and chewing. Blood analysis showed improved protein levels, liver and kidney function, and immune response. Additionally, PEL supplementation regulated stress and hormone levels, while gene expression analysis revealed upregulation of growth- and antioxidant-related genes. Notably, 4 g/day of PEL significantly improved the health and performance of pregnant does.

## 1. Introduction

Zaraibi goats, also known as Egyptian Nubians, are a well-established breed in Egypt and the Near East. Their adaptability to diverse environments, combined with their strong reproductive traits and high-quality milk and meat production, make them a valuable livestock species in the region. However, pregnancy is a critical period for the dam, marked by significantly increased energy and oxygen demands to support fetal growth [1]. This heightened metabolic activity can elevate the risk of oxidative stress in both the dam and the developing fetus. Additionally, pregnancy represents a unique immunological state in which the maternal immune system must perform a dual role: protecting both the dam and fetus from infections while also facilitating the physiological adaptations necessary for a successful pregnancy [2]. As a result, there is growing interest among livestock producers in using phytogenic natural products as feed additives, offering a sustainable approach to enhancing animal health during critical periods such as pregnancy [3,4].

Feed additives during late pregnancy in goats are essential for supporting fetal development, enhancing maternal health, and optimizing nutrient utilization to meet the increased energy and nutritional demands of this critical period [3]. Various feed additives are used during this time. For example, supplementation with *Lactobacilli* (LAB)-based direct-fed microbials can modulate the microbial populations of the digestive tract, potentially enhancing fermentation efficiency and nutrient uptake in transition ruminants [5,6]. Furthermore, research consistently demonstrates the benefits of LAB supplementation in ruminants. These microorganisms help maintain gut microbiome balance, enhance digestion, reduce inflammation, and improve feed efficiency [5,6,7,8]. Additionally, LAB provide immune support, exhibit antioxidant activity, and mitigate stress-related gut disturbances during critical periods such as weaning or transportation, ultimately enhancing overall animal productivity [9,10]. Similarly, phytochemicals (PFAs)—naturally occurring bioactive compounds produced by plants—have been shown to enhance feed utilization, improve animal health, and boost productivity [11]. The diverse biological activities of PFAs, including their antimicrobial properties and lower risk of antimicrobial resistance, make them a promising alternative to antibiotics, promoting livestock health and performance in an environmentally friendly manner [12]. Given the promising individual effects of PFAs and LAB, their combined synergistic potential as feed additives warrants further investigation [13].

While the benefits of PFAs and LAB have been extensively studied in various animal species, their combined effects—particularly in pregnant does—remain largely unexplored. The synergistic interaction between LAB and PFAs may help animals navigate the transition period by addressing digestive, immune, and metabolic challenges. LAB stabilize the gut microbiome, enhancing nutrient utilization and fermentation efficiency, while PFAs, with their antimicrobial and bioactive properties, further support gut health by suppressing harmful pathogens. Both LAB and PFAs exhibit anti-inflammatory effects and strengthen immune function, mitigating the physiological and metabolic stress common during late pregnancy. Together, they may optimize energy metabolism, enhance stress resilience, and improve overall health and productivity during this critical phase. Therefore, this study aimed to evaluate the effects of combining plant-derived PFAs with *lactobacilli* (PEL) on Zaraibi goat performance during late pregnancy, focusing on feed intake, ingestive behavior, nutrient digestion, rumen function, health responses, and gene expression.

## 2. Materials and Methods

The experiment was conducted at the Experimental Research Station Farm in El-Serw, affiliated with the Animal Production Research Institute, Agricultural Research Center, Ministry of Agriculture, Egypt, in collaboration with the Animal Production Department, Faculty of Agriculture, Mansoura University, Egypt. The study was approved by the Scientific Research Ethics Committee of the Faculty of Agriculture, Mansoura University, and the Animal Production Research Institute, Agricultural Research Center, Egypt (protocol code 551429—20 September 2023).

### 2.1. The Experimental PEL Feed Additive

According to the manufacturer, the experimental PEL (Digeston-Green^®^, P.G.E., Mitterlabill, Austria) was derived from herbal extracts, including oregano, anise, thyme, eucalyptus, and rosemary, and was combined with lactic acid bacteria (10^6^ CFU/g dry matter). The primary volatile bioactive PFAs were carvacrol (83.0 mg/kg), trans-anethole (98.25 mg/kg), and thymol (45.75 mg/kg). Additionally, we analyzed the non-volatile PFAs in the PEL using gas chromatography–mass spectrometry (GC–MS) with a Thermo Scientific TRACE-1300 series gas chromatograph (Thermo Fisher Scientific Inc., Waltham, MA, USA) equipped with a DB-5 capillary column (30 m × 0.32 mm i.d., 0.25 μm film thickness) and coupled to a Triple Quadrupole Mass Spectrometer (TSQ 8000 Evo, Thermo Fisher Scientific, Waltham, MA, USA). The identification of PEL components was based on retention index data and mass spectral analysis using the Mainlib library [14]. A total of 25 components were identified by GC-MS, with the major compounds being elaidic acid isopropyl ester, cis-7,cis-11-hexadecadien-1-yl acetate, ascorbic acid, and D-glucopyranosiduronic acid (Table 1).

### 2.2. Animals and Management

A total of 30 Zaraibi does (30.9 ± 0.37 kg body weight, 3 to 3.5 years old, and 90 days pregnant) were randomly selected from a herd of 200 goats and assigned to 3 experimental groups in a Completely Randomized Design (CRD). The animals were homogeneous in key physiological traits, minimizing the need for blocking. The does were managed under a semi-intensive system and housed in shaded, semi-open pens with concrete flooring to protect against environmental stress while ensuring adequate ventilation.

Pregnancy status was determined using transrectal ultrasonography with a real-time B-mode scanner. All ultrasound scans were recorded on high-quality videotape and stored digitally for later analysis. Before examination on days 15 and 35 post-mating, goats were fasted for approximately 8 h. During transabdominal ultrasonography, the transducer was placed on both sides of the inguinal region after applying carboxymethyl cellulose gel (Echo gel, IBE Co., Cairo, Egypt) to enhance contact and image resolution.

The litter size of each doe was recorded, and only pregnant does carrying twin fetuses were selected to ensure uniformity in fetal load. Since litter size influences maternal nutrient requirements and physiological adaptations during pregnancy, this selection minimized variability in metabolic responses. After 90 days of pregnancy, all does were stratified into three groups (10 does per group) and assigned to an experimental basal diet supplemented with the PEL mixture at 0 g (control), 2 g, or 4 g/day.

Supplementation using the PEL began on day 90 of pregnancy and continued for 60 days. The experimental basal diet was formulated to meet NRC [15] recommendations for lactating does, with its ingredients and chemical composition detailed in Table 2. The PEL additive was mixed daily with the concentrate before feeding and then blended with the roughage. Diets were offered individually to each doe in two equal portions at 08:00 and 15:00 h, with free access to fresh water.

### 2.3. Feed Intake

Throughout the experiment, feed intake was monitored by measuring the feed offered and refused for each doe. Body weight was recorded at the start and every two weeks before morning feeding. Feed amounts were adjusted based on weight changes to optimize utilization, ensuring refusals did not exceed 10% of daily dry matter intake.

### 2.4. Digestibility Trial and Chemical Analyses

During the final week of pregnancy (day 143), daily feed and fecal samples were collected from each doe and stored at −20 °C for later analysis. Fresh fecal samples (~70 g per doe) were obtained before morning feeding. At the end of the experiment (day 150), all samples were pooled per doe, dried at 70 °C for 48 h in a forced-air oven, ground, and analyzed for key nutrients (dry matter, ash, crude protein, crude fiber, and ether extract) following standard methods [16]. Nitrogen-free extract was calculated from the analysis. Digestion coefficients and feeding values, including total digestible nutrients and digestible crude protein, were determined for each group using established guidelines [17].

### 2.5. Rumen Fermentation Parameters

At the end of the experiment, ruminal fluid was collected from each doe three hours after morning feeding using a flexible tube. After discarding the first 50 mL, 100 mL of ruminal fluid was collected and filtered. Ruminal pH was measured immediately using a digital pH meter (Adwa AD11, Szeged, Hungary). A 5 mL subsample was preserved for ammonia-nitrogen (NH_3_-N) analysis [18]. For volatile fatty acid (VFA) analysis, 0.8 mL of ruminal fluid was mixed with 0.2 mL of a 250 g/L metaphosphoric acid solution and centrifuged at 15,000 rpm for 20 min at 4 °C (K1015 Micro Prime; Centurion Scientific Ltd., Chichester, UK). VFA concentrations were determined [19] using gas chromatography (Thermo Fisher Scientific, TRACE 1300, Milan, Italy) with an AS3800 autosampler and an HP-FFAP capillary column (19091F-112; 0.320 mm OD, 0.50 µm ID, 25 m length; J&W Agilent Technologies, Santa Clara, CA, USA).

### 2.6. Ingestive Behavior

Ingestive behavior was assessed at 150 days of gestation through continuous 24 h visual observations. Feeding, ruminating, and idling times were recorded following the methodology of Johnson and Combs [20]. Total chewing time was calculated by summing feeding and rumination times [21]. Three trained observers collected data using digital stopwatches, ensuring accuracy and consistency. Observations were conducted in real-time at predetermined intervals following a standardized protocol to minimize subjective bias. While no video surveillance was used, assessments focused on specific behavioral patterns relevant to the study objectives. Dry matter feed efficiency and dry matter rumination efficiency (g DM/h) were calculated using equations adapted from Polli et al. [22] and Carvalho et al. [23].

### 2.7. Blood Sampling

On day 150 of pregnancy, before the morning feeding, blood samples were collected from seven does per group. Each sample was divided into three subsamples. One subsample (10 mL) contained an anticoagulant for a complete blood count (CBC), which included white blood cell count, red blood cell count, hemoglobin, hematocrit, platelet count, mean corpuscular volume (MCV), mean corpuscular hemoglobin (MCH), and lymphocyte, neutrophil, monocyte, and eosinophil concentrations.

The second subsample (10 mL) was allowed to clot at room temperature for 20 min, then centrifuged at 3000 rpm for 20 min to separate the serum, which was stored at −20 °C for later analysis. The serum was used to determine a biochemical profile, including total protein, albumin, alanine aminotransferase (ALT), aspartate aminotransferase (AST), urea, creatinine, triglycerides, cholesterol, high-density lipoprotein (HDL), low-density lipoprotein (LDL), very-low-density lipoprotein (VLDL), glucose, lipase, and amylase. Analyses were conducted using commercial test kits (Spectrum Diagnostics, Cairo, Egypt) and a CHEM 7 spectrophotometer (ERBA, Mannheim, Germany). Globulin concentration was calculated by subtracting albumin from total protein. Additionally, serum cortisol, immunoglobulin G (IgG), and immunoglobulin M (IgM) were measured using commercial ELISA kits (CUSABIO Biotech, Wuhan, China). The third subsample (10 mL), also containing an anticoagulant, was used for RNA extraction.

### 2.8. Total RNA Extraction, Reverse Transcription, and Quantitative Real Time PCR

Total RNA was extracted from blood samples using Trizol reagent (RNeasy Mini Kit, Cat. No. 74104), following manufacturer’s protocol. The concentration and purity of the isolated RNA were assessed using a NanoDrop^®^ ND-1000 spectrophotometer (Thermo Fisher Scientific, Wilmington, DE, USA). Complementary DNA (cDNA) was synthesized from each sample according to the manufacturer’s instructions (Thermo Fisher, Cat. No. EP0441). Quantitative RT-PCR was performed using SYBR Green PCR Master Mix (2× SensiFast™ SYBR, Bioline, Cat. No. Bio-98002, London, UK) to evaluate the expression of immunity and antioxidant-related genes. Real-time PCR was conducted using the Quantitect SYBR Green PCR Kit (Cat. No. 204141, Qiagen, Hilden, Germany) to quantify relative mRNA levels. Primer sequences were designed based on the Capra hircus genome (GenBank database) and are listed in Table 3.

The housekeeping gene β-actin was used as a constitutive control for normalization. The reaction mixture (25 µL total volume) contained 3 µL of total RNA, 4 µL of 5× TransAmp buffer, 0.25 µL of reverse transcriptase, 0.5 µL of each primer, 12.5 µL of 2× Quantitect SYBR Green PCR Master Mix, and 8.25 µL of RNase-free water. The thermal cycling program included the following: reverse transcription at 50 °C for 30 min, initial denaturation at 94 °C for 10 min, followed by 40 cycles of denaturation at 94 °C for 15 s, annealing at gene-specific temperatures, and extension at 72 °C for 30 s. A melting curve analysis was performed at the end of the amplification phase to confirm product specificity (Table 3). Relative gene expression was calculated using the 2^−ΔΔCt^ method, with β-actin as the reference gene [24].

### 2.9. Statistical Analysis

The data were analyzed using a one-way ANOVA, with dietary supplementation as a fixed factor. The statistical model used was Yij = µ + Pi + Eij, where Yij represents the individual observation, µ is the overall mean, Pi is the supplementation effect, and Eij is the random error. Analyses were conducted using SAS v.9.3 (SAS Institute Inc., Cary, NC, USA). Linear and quadratic orthogonal polynomial contrasts were applied to assess supplementation-level responses. Each doe was considered an experimental unit. Least square means and their standard errors were reported, with statistical significance set at *p* < 0.05.

## 3. Results

### 3.1. Feed Intake, Digestibility, and Ruminal Parameters

Nutrient intake in Zaraibi does increased linearly (*p* ≤ 0.01) with PEL supplementation (Table 4). Daily dry matter intake was enhanced by 4.2% with 2 g PEL/day and 7.1% with 4 g PEL/day compared to the control group. Similarly, total digestible nutrient intake increased by 3.1% and 7.3% for the 2 g and 4 g PEL/day treatments, respectively. Digestible crude protein intake also increased by 3.4% and 7.4% with 2 g and 4 g PEL/day, respectively, relative to the control group.

The nutrient digestibility of dry matter, crude fiber, nitrogen-free extract, and total digestible nutrients increased linearly (*p* ≤ 0.01) with PEL supplementation. Additionally, the digestibility of organic matter, crude protein, ether extract, and digestible crude protein showed both linear and quadratic improvements (*p* < 0.05).

At 150 days of pregnancy, ruminal parameters were influenced by PEL supplementation (Table 4). Ruminal pH and ammonia-N concentrations decreased linearly (*p* < 0.05) with PEL treatment, with the lowest ammonia-N levels observed in the 4 g PEL/day group, followed by the 2 g PEL/day group (8.4% and 4.7% lower than the control, respectively; *p* < 0.001). Acetic acid concentrations increased both linearly and quadratically (*p* < 0.01), while propionic acid exhibited a quadratic increase (*p* < 0.01) in response to PEL supplementation. In contrast, butyric acid concentrations decreased linearly (*p* < 0.01).

### 3.2. Ingestive Behavior

**Figure 1:**
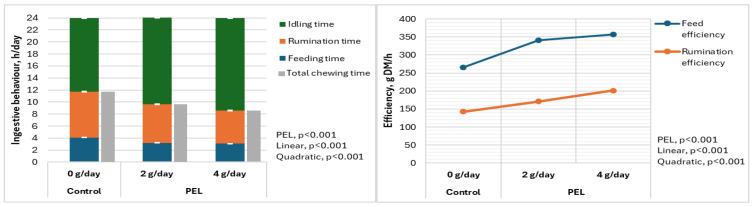
Effect of experimental blend of phytochemicals and lactic acid bacteria feed additive (PEL) on ingestive behavior of Zaraibi does.

Figure 1 shows that the PEL affected all aspects of does’ ingestive behavior (linear, *p* < 0.001; quadratic, *p* < 0.001). Compared to the control group, feeding and rumination time per day decreased by 24.4% and 28.1% for 4 g PEL/day, respectively, while idling time increased by 20.1% (*p* < 0.001).

Additionally, total chewing time per day was reduced by 26.7% in the 4 g PEL/day group compared to the control. Moreover, the 4 g PEL/day improved both feed and rumination efficiency by 27.8% and 28.9%, respectively (linear, *p* < 0.001; quadratic, *p* < 0.001).

### 3.3. Blood Parameters

The hematological parameters of the does at 150 days of pregnancy were influenced by PEL supplementation (Table 5). Hemoglobin, red blood cell count, hematocrit, platelet count, and neutrophil levels increased linearly (*p* < 0.001) with PEL inclusion. The highest values for these parameters were observed in the 4 g PEL/day group, followed by the 2 g PEL/day group. The mean corpuscular hemoglobin (MCH), mean corpuscular volume (MCV), and mean corpuscular hemoglobin concentration (MCHC), along with white blood cell, lymphocyte, monocyte, and eosinophil counts, decreased linearly (*p* < 0.001) with PEL supplementation. The lowest values for these parameters were observed with 4 g/day of PEL, followed by 2 g/day of PEL. Both red and white blood cell counts were influenced linearly and quadratically (*p* ≤ 0.005) by PEL supplementation in the does’ diet.

The supplementation of PEL in the does’ diet affected blood serum parameters at 150 days of pregnancy (Table 6). Serum total protein, albumin, globulin, HDL, lipase, amylase, and thyroxine (T4) increased linearly (*p* < 0.001) with PEL supplementation, with the highest values observed at 4 g/day of PEL, followed by 2 g/day of PEL. In contrast, the albumin/globulin ratio, aspartate aminotransferase (AST), alanine aminotransferase (ALT), urea, creatinine, total cholesterol, triglycerides, LDL, VLDL, glucose, cortisol, and triiodothyronine (T3) decreased linearly (*p* < 0.001) with PEL supplementation. All serum parameters were also affected quadratically (*p* ≤ 0.04) by PEL inclusion, except for globulin, the albumin/globulin ratio, LDL, and T3 concentrations.

Adding the PEL to the diet of Zaraibi does affected blood serum immunity, antioxidant, and inflammation markers (Table 7). IgG and IgM levels increased (linear, *p* < 0.001; quadratic, *p* < 0.001) with PEL supplementation, with the highest values observed at 4 g/day. Total antioxidant capacity, glutathione peroxidase (linear, *p* < 0.001; quadratic, *p* < 0.001), and catalase activity (linear, *p* < 0.001) also increased with PEL addition. In contrast, malondialdehyde levels decreased (linear, *p* < 0.001; quadratic, *p* ≤ 0.004), with the lowest values observed at 4 g/day. PEL supplementation also reduced interleukin-1 beta (IL-1β) (linear, *p* < 0.001) and interleukin-6 (IL-6) (linear, *p* < 0.001; quadratic, *p* < 0.001), with the lowest concentrations at 4 g/day.

### 3.4. Gene Expression

**Figure 2:**
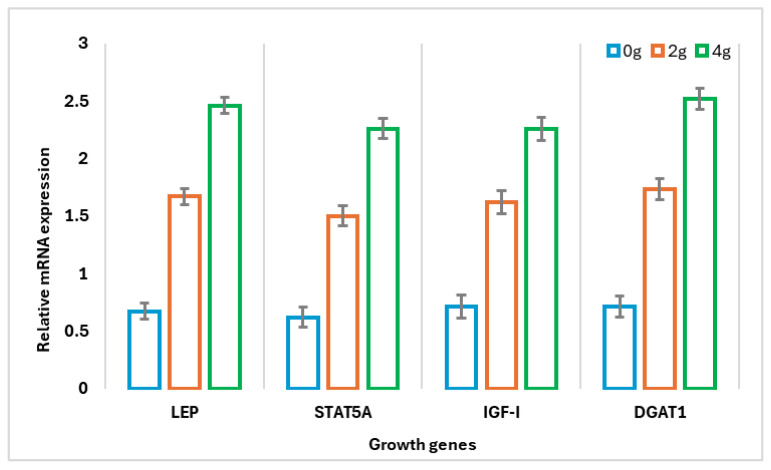

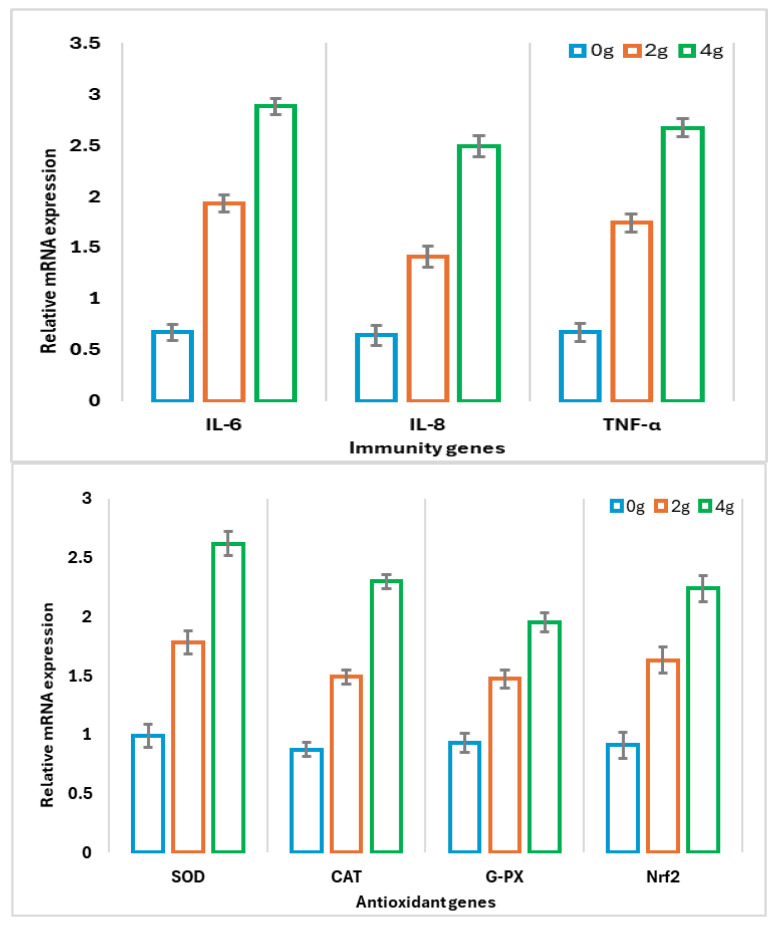
Effect of experimental blend of phytochemicals and lactic acid bacteria feed additive (PEL) on relative expression patterns of growth, immunity, and antioxidant marker genes in Zaraibi does. LEP, leptin; STAT5A, signal transducer and activator of transcription 5A; IGF-I, insulin-like growth factor 1; DGAT1, diacylglycerol o-acyltransferase 1; IL, interleukin; TNF-α, tumor necrosis factor alpha; SOD, superoxidedismutase; CAT, catalase; GPx, glutathione peroxidase; NrF2, nuclear factor erythroid 2.

PEL supplementation affected the relative expression of growth-related, immunity, and antioxidant marker genes at 150 days of pregnancy (Figure 2). Supplementation with 4 g/day of PEL increased the expression of growth-related genes (LEP, STAT5A, IGF-I, and DGAT1) by 72.8%, 72.6%, 68.6%, and 71.8%, respectively, compared to the control group (*p* < 0.001). Similarly, immunity-related genes (IL-6, IL-8, and TNF-α) showed a linear increase of 76.7%, 74.3%, and 74.9% (*p* < 0.001), respectively, with 4 g/day of PEL. Additionally, the expression of antioxidant genes (SOD, CAT, GPx, and Nrf2) increased by 62.2%, 62.2%, 52.3%, and 59.4% (*p* < 0.001), respectively, compared to the control group.

## 4. Discussion

The experimental PEL was selected for its diverse range of naturally occurring beneficial compounds derived from multiple plant sources. Many of these compounds have been shown to possess various health-promoting properties, including antioxidant, anti-inflammatory, and antimicrobial effects. Combining different plant-based compounds can create a powerful synergy, enhancing their bioactive properties [3,25]. On the other hand, LAB bacteria are widely recognized for their significant role in animal nutrition. Numerous studies have shown that LAB supplementation in ruminants provides various health benefits, including gut microbiota regulation, enhanced intestinal motility, microbial balance maintenance, inflammation reduction, and improved overall intestinal function [5,6,7,26].

The GC-MS analysis of the experimental PEL reveals a diverse array of compounds, including fatty acids, esters, sterols, vitamins, and other organic molecules. This complex composition suggests a broad spectrum of potential biological activities, which may contribute to the beneficial effects observed in previous studies. The high abundance of compounds like cis-7, cis-11-hexadecadien-1-yl acetate, and elaidic acid isopropyl ester highlights the significant contribution of these specific components to the overall composition of the blend. Additionally, ascorbic acid 2,6-dihexadecanoate and α-D-glucopyranosiduronic acid derivatives were also present in relatively high concentrations. The presence of biologically active compounds such as linoleic acid, α-tocopherol (vitamin E), β-carotene, and α-sitosterol further supports the potential for a wide range of physiological effects. Several studies have highlighted the significant pharmacological and biological effects of the detected components found in PEL, demonstrating properties such as immunomodulatory, antibacterial, antioxidant, and anti-inflammatory activities [3,14].

This study demonstrated that PEL supplementation significantly influenced feed intake, nutrient digestibility, and ruminal fermentation in does. The improved feed efficiency observed in PEL-supplemented groups was likely attributed to the bioactive compounds present in the phytogenic components. The higher feed intake observed in the experimental groups was likely due to the improved digestibility of nutrients, which promoted diet turnover and further stimulated feed consumption [27,28]. Phytogenic compounds, particularly in combination with LAB bacteria, have been shown to improve feed palatability and enhance nutrient breakdown and absorption [27,29,30].

Previous studies have demonstrated that PFAs may increase the digestibility of crude protein and organic matter, leading to better nutrient utilization and faster rumen turnover, which in turn encourages higher feed intake. For example, Liu et al. [31] found that supplementing prebiotics and essential oils improved dry matter intake and feed efficiency, while Hashemzadeh et al. [32] reported that herb supplementation in heat-stressed lambs led to higher feed intake and improved lamb performance. These findings support the notion that enhanced nutrient digestibility plays a key role in driving increased feed consumption. Moreover, the PFAs of the PEL have been suggested as promising alternatives for manipulating the rumen microbial population to optimize energy and protein utilization in ruminants [33,34]. These additives have demonstrated antimicrobial properties within the rumen, exhibiting broad-spectrum activity against various microorganisms, including both Gram-negative and Gram-positive bacteria [35], similar to the effects observed with antibiotics. Therefore, a blend of phytochemicals and lactic acid bacteria can modulate the composition and function of the gut microbiota, promoting a healthier gut environment and reducing the risk of infections [7,11,36].

The observed changes in the ruminal parameters of pregnant does supplemented with the PEL provide valuable insights into its effects on rumen function. The significant decrease in ruminal ammonia-N concentration with increasing PEL levels is particularly noteworthy. By reducing ammonia-N and slightly decreasing ruminal pH, PEL supplementation likely fosters a more balanced and efficient microbial ecosystem within the rumen. This improved microbial activity is further evidenced by the increase in total volatile fatty acid concentration, particularly acetic and propionic acid, with 4 g/day of PEL. Additionally, PELs have demonstrated effectiveness in maintaining ruminal pH and enhancing ruminal fermentation, particularly in high-grain diets [37]. This beneficial effect is attributed to their ability to modulate ruminal microbial populations. For instance, PFAs such as plant-derived alkaloids [38], β-sitosterol [39], tannic acid [40], and phenolic plant extracts [41] have been shown to inhibit the growth of lactate-producing bacteria while promoting the growth of beneficial bacteria that utilize lactic acid. This microbial shift ultimately contributes to increased ruminal pH, reduced lactate accumulation, and decreased lipopolysaccharide levels. Our results emphasize the dynamic nature of ruminal fermentation in response to external interventions and highlight the potential benefits for animal health, including improved nutrient absorption, enhanced performance, and strengthened immune responses.

The improved goats’ dry matter digestibility observed in the PEL groups may be attributed to the presence of bioactive compounds in the PEL extracts combined with the LAB, which enhanced ruminal fermentation parameters and consequently improved nutrient digestion and absorption. Moreover, the synergistic interaction between the experimental PFAs in the phytogenic plant extracts and lactic acid bacteria can positively influence the gut microbiota, thereby promoting digestive efficiency and nutrient uptake in the does. This combination may foster beneficial bacterial symbiosis and the growth of fermentative microorganisms, leading to enhanced nutrient digestion. Although the limited number of experimental does is a limitation of this study, the positive impact of blended phytogenic plant extracts and lactic acid bacteria on apparent nutrient digestibility is likely to be accompanied by improvements in animal health status.

Limited research has evaluated the impact of PFAs and/or LAB on ingestive behavior in ruminants, particularly in late-pregnant does. However, the observed reduction in feeding and rumination time, along with an increase in idling time, suggests that PEL supplementation enhances feed digestibility in does. This enhanced digestibility likely results in faster nutrient absorption, enabling the animals to meet their nutritional requirements with less feeding and rumination. Consequently, the reduction in total chewing time indicates that the PEL-supplemented diet may require less extensive oral processing, potentially due to enhanced rumen fermentation. This, in turn, contributes to significant improvements in feed and rumination efficiency [42], suggesting that goats extract more nutrients with less effort, which has significant implications for animal productivity and overall health. Furthermore, our findings are consistent with previous research showing that rumination cycles are influenced by factors such as feed structure, meal frequency, and feed intake [43,44]. Moreover, diets with lower cell wall contents and higher starch contents are associated with decreased chewing times and faster food passage [45]. Junior et al. [35] observed that increasing dietary levels of *Arnica montana* essential oils (EOs) in lambs resulted in a linear increase in meal frequency and overall feeding time. However, feeding rate and rumination time exhibited a quadratic response to increasing EO levels. In contrast, Da Silva et al. [46] and Geron et al. [47] found that phytogenic feed additives did not significantly affect ingestive behavior patterns in lambs. These results, in conjunction with our observation of reduced feeding and rumination times in does treated with the PEL, suggest that the phytogenic levels used in some studies may not have significantly altered the rumen environment. This could have limited their impact on key factors influencing ingestive behavior, such as rate of passage and feed retention time [47].

Blood hematological and metabolite analyses are valuable tools for diagnosing disorders, assessing physiological status, and evaluating animal well-being. The observed increases in hemoglobin, red blood cell count, hematocrit, and platelet count with PEL supplementation suggest improved erythropoiesis and overall hematopoiesis. These findings indicate that PEL may enhance iron absorption and utilization, leading to the improved oxygen-carrying capacity of blood. Moreover, the increase in neutrophils, which serve as the first line of defense against infections [48], suggests that PEL supplementation enhances the immune response of the does. Conversely, the decrease in lymphocytes, monocytes, and eosinophils may reflect a shift in the immune system toward a more anti-inflammatory state [49,50], which is generally beneficial during pregnancy.

Elevated levels of total protein, albumin, and globulin, which are crucial for nutrient transport, immune function, and fluid balance, suggest enhanced protein metabolism. This is further supported by the improved liver function, as indicated by increased HDL- cholesterol and decreased liver enzymes (AST and ALT). Concurrently, PEL supplementation appears to positively influence lipid metabolism by lowering serum glucose, triglycerides, and cholesterol and potentially improving insulin sensitivity, an essential factor during the increased metabolic demands of pregnancy. Interestingly, PEL supplementation appears to modulate thyroid hormone levels, with increased T4 and decreased T3. While the precise implications of this require further investigation, it suggests potential adaptations in thyroid function to support the physiological demands of pregnancy. The quadratic effects observed in most serum parameters highlight the complex interplay of these interactions, suggesting that an optimal PEL supplementation level likely exists to maximize its beneficial effects on biochemical parameters. However, Waqas et al. [51] reported elevated total protein, serum globulin, and blood urea nitrogen levels in cows fed a plant-based additive mixture, indicating improved protein metabolism and nutritional status. In heat-stressed dairy calves, supplementation with a PFA mixture had a minimal impact on growth performance but effectively reduced inflammatory markers [52]. Similarly, supplementing heat-stressed lambs with a mixture of clove, rosemary, turmeric, and cinnamon buds slightly increased cholesterol levels while reducing creatinine [32]. Moreover, LAB bacteria have been shown to modulate the immune system, lower serum cholesterol, and exhibit potential anti-tumor activity [53,54].

The PEL supplementation exerted multifaceted benefits on the health of pregnant does, including enhanced immunity, improved antioxidant defense, and a modulated inflammatory response. The observed increase in total antioxidant capacity, along with the elevated activities of key antioxidant enzymes such as glutathione peroxidase and catalase, suggests a strengthened defense against oxidative stress.

This is vital, as oxidative stress can negatively impact maternal and fetal health during pregnancy [55,56]. The decrease in malondialdehyde (MDA), a biomarker of lipid peroxidation, further supports the notion of reduced oxidative stress within the does. Moreover, the significant decreases in pro-inflammatory cytokines IL-6 and IL-1β suggest that PEL supplementation effectively modulates the inflammatory response. This is crucial, as excessive inflammation can have detrimental effects on both the does and the developing fetus. However, previous studies suggest that PFAs play a crucial role in enhancing the immune function of ruminants by modulating immune cell function, stimulating antibody production, and improving overall immune responsiveness. These findings are supported by other studies demonstrating the immunomodulatory effects of various PFAs [11,57]. For example, Molosse et al. [58] observed increased IgA and IgG levels in Holstein’s calves supplemented with a combination of curcumin and a phytogenic blend. Bostami et al. [59] reported enhanced lymphocytic proliferation and increased IgG levels in post-weaned bull calves supplemented with a mixture of *Terminalia bellirica*, *Emblica officinalis*, and *Terminalia chebula*. Additionally, Shedeed et al. [60] observed increased white blood cell counts and IgA levels in Barki ewes supplemented with propolis. Guo et al. [61] reported that licorice extract supplementation in Karakul sheep elevated total antioxidant capacity and improved concentrations of IgA and IgG.

Supplementation with PFAs demonstrates potent antioxidant and anti-inflammatory activities in ruminants through multiple mechanisms. These include scavenging free radicals, stimulating antioxidant enzymes such as superoxide dismutase (SOD) and glutathione peroxidase (GPx) [62], and modulating mitochondrial function [63]. Enzymes such as SOD, GPx, and catalase (CAT) play a crucial role in protecting both the does and fetuses from oxidative stress during pregnancy by neutralizing harmful free radicals [64]. Among these, polyphenols—abundant in many PFAs—are particularly important due to their strong electron-donating capacity, which enables effective free radical neutralization [11,65]. Research has consistently shown that PFAs derived from various plant sources, including oregano, cinnamon, turmeric, Echinacea purpurea, and saffron, significantly enhance antioxidant status in ruminants. These effects manifest through increased antioxidant enzyme activity, reduced oxidative stress markers, and improved overall health and performance [59,66,67,68]. In general, the enhanced antioxidant activity of the experimental PFAs primarily stems from their phenolic compounds, which function as potent antioxidants by donating hydrogen atoms and electrons to neutralize free radicals, thereby disrupting oxidative chain reactions within tissues [11]. Similarly, recent research on the antioxidant mechanisms of LAB indicates that LAB’s intrinsic antioxidant system, combined with bioactive compounds produced during substrate fermentation, confers enhanced antioxidant activity [8].

The observed upregulation of growth-related genes, including LEP, STAT5A, IGF-I, and DGAT1, suggests that PEL supplementation stimulates anabolic processes, fostering tissue growth and development in pregnant does. This effect may also help mitigate the risk of weight loss due to negative energy balance, which often occurs after parturition. At the same time, PEL supplementation appears to modulate the immune response, as evidenced by the increased expression of immune-related genes such as IL-6, IL-8, and TNF-α. While an increase in these pro-inflammatory cytokines might seem paradoxical, it is crucial to recognize that a well-regulated inflammatory response plays a vital role during pregnancy. Furthermore, the upregulation of antioxidant genes, including SOD, GPx, Nrf2, and CAT, indicates a marked enhancement of the antioxidant defense system [69]. This is of paramount importance during pregnancy, as oxidative stress can have detrimental effects on both the mother and the developing fetus [55]. A robust antioxidant defense system is essential for mitigating oxidative damage and ensuring optimal fetal development [70].

Generally, the observed enhancements in nutrient digestibility, ingestive behavior, blood biochemical parameters, and immune and antioxidant responses suggest a potential dose-dependent effect of PEL supplementation in pregnant Zaraibi goats. The positive effects observed at the 4 g/day supplementation level suggest that this dosage may be optimal for maximizing these beneficial responses under the conditions of this study.

## 5. Conclusions

The experimental findings suggest that PEL supplementation creates a more favorable physiological environment for pregnant Zaraibi does by enhancing immune defenses, boosting antioxidant capacity, reducing the metabolic burden of pregnancy, and mitigating the detrimental effects of inflammation. Moreover, by enhancing feed digestibility and ingestive behavior, PEL may contribute to healthier dams, stronger offspring, and improved economic returns for livestock producers. These results indicate that supplementing Zaraibi does with 4 g/day of PEL could be an effective strategy for enhancing health and productivity during pregnancy.

## Figures and Tables

**Table 1:** Constituents identified by gas chromatography and mass spectrometry analysis of experimental blend of phytochemicals and lactic acid bacteria (PEL).

Peaks	Compounds	Retention Time, min	Peak Area (%)	Molecular Weight	Molecular Formula
1	á-d-Glucopyranosiduronic acid, 3-(5-ethylhexahydro-2,4,6-trioxo-5-pyrimidin yl)-1,1-dimethylpropyl 2,3,4-tris-*O*-(trimethylsilyl)-, methyl ester	4.22	9.99	648	C_27_H_52_N_2_O_10_Si_3_
2	4-Hexyl-1-(7-methoxycarbonylheptyl)bicyclo[4.4.0]deca-2,5,7-triene	4.39	2.86	372	C_25_H_40_O_2_
3	Decanoic acid, 1,1a,1b,4,4a,5,7a,7b,8,9-decahydro-4a,7b-dihydroxy-3-(hydroxymethyl)-1,1,6,8-tetramet hyl-5-oxo-9aH-cyclopropa[3,4]benz[1,2-e]azulene-9,9a-diyl ester, [1aR-(1aà,1bá,4aá,7aà,7bà,8à,9á,9aà)]	25.41	1.45	672	C_40_H_64_O_8_
4	Pentadecanoic acid, 14-methyl-, methyl ester	25.45	1.01	270	C_17_H_34_O_2_
5	L-Valine, N-[2-(chloroimino)-3-methyl-1-oxobutyl]-	25.52	1.30	248	C_10_H_17_ClN_2_O_3_
6	l-(+)-Ascorbic acid 2,6-dihexadecanoate	31.66	12.19	652	C_38_H_68_O_8_
7	à-d-Galactopyranose, 6-*O*-(trimethylsilyl)-, cyclic 1,2:3,4-bis(butylboronate)	33.64	4.78	384	C_17_H_34_B_2_O_6_Si
8	Linoleic acid ethyl ester	34.21	1.28	308	C_20_H_36_O_2_
9	cis-7,cis-11-Hexadecadien-1-yl acetate	35.39	29.13	280	C_18_H_32_O_2_
10	Elaidic acid, isopropyl ester	35.50	14.38	324	C_21_H_40_O_2_
11	Octadecanal, 2-bromo	36.16	2.36	346	C_18_H_35_BrO
12	9-Octadecenoic acid, (2-phenyl-1,3-dioxolan-4-yl)methyl ester, cis	37.08	2.13	444	C_28_H_44_O_4_
13	Milbemycin B, 6,28-anhydro-15-chloro-25-isopropyl-13-dehydro-5-*O*-demethyl-4-methyl-	41.16	1.21	590	C_33_H_47_ClO_7_
14	à-d-Xylopyranoside, methyl-2,3,4-tris-*O*-[9 borabicyclo[3.3.1]non-9-yl]	41.64	1.26	524	C_30_H_51_B_3_O_5_
15	Ursa-9(11),12-dien-28-oic acid, 3-(acetyloxy)-, methyl ester, (3á)-	45.86	4.22	510	C_33_H_50_O_4_
16	Betulin	50.64	0.84	442	C_30_H_50_O_2_
17	Ursodeoxycholic acid	51.13	1.18	392	C_24_H_40_O_4_
18	9,12,15-Octadecatrienoic acid, 2-phenyl-1,3-dioxan-5-yl ester	51.18	0.64	440	C_28_H_40_O_4_
19	Milbemycin B,5-demethoxy-5-one-6,28-anhydro-25-ethyl-4-methyl-13-chloro-oxime	51.22	0.39	589	C_32_H_44_ClNO_7_
20	á Carotene	51.26	0.80	536	C_40_H_56_
21	Sulfadiazine	51.34	0.59	250	C_10_H_10_N_4_O_2_S
22	á-Sitosterol	51.51	2.21	414	C_29_H_50_O
23	.psi.,.psi.-Carotene	52.04	1.52	600	C_42_H_64_O_2_
24	25-Norisopropyl-9,19-cyclolanostan-22-en-24-one, 3-acetoxy-24-phenyl-4,4,14-trimethyl-	54.44	0.77	516	C_35_H_48_O_3_
25	2,2′-Methylenebis[3,4,6-trichloroanisole]	54.53	1.51	432	C_15_H_10_C_l6_O_2_

**Table 2:** Ingredients and nutrient composition of basal diet.

Item	% (Dry Matter Bases)
Ingredients	
Egyptain Berssem (*Trifolium alexandrinum*) clover hay	25.0
Wheat straw	25.0
Yellow corn	22.5
Un-decorticated cottonseed meal	12.5
Wheat bran	10.0
Rice bran	2.0
Molasses	1.5
Limestone	1.0
Vitamin and minerals mixture *	0.5
Chemical composition	
Dry matter	91.0
Organic matter	89.3
Crude protein	11.9
Ether extract	25.0
Nitrogen-free extract	2.53

* Containing, per kg of premix: 500,000 IU vitamin A, 100,000 IU vitamin D3, 1500 mg vitamin E, 180 g calcium, 80 g phosphorus, 20 g magnesium, 50 g sodium, 3000 mg zinc, 2000 mg manganese, 500 mg copper, 50 mg iodine, 10 mg selenium, and 20 mg cobalt.

**Table 3:** Oligonucleotide primer sequences, accession numbers, annealing temperatures and PCR product sizes of immune and antioxidant genes used in real-time PCR.

Gene	Primer	Product Length (bp)	Annealing Temperature (°C)	Accession Number
*Leptin*	F5′-CAGTCCGTCTCCTCCAAACA-3′ R5′-CGGAGGTTCTCCAGGTCATT-3′	170	60	EU158187.1
*STAT5A*	F5′-TGGGGCCTTCCTGTAGTAAC-3′ R5′-CGGGGATATTCCAGCCCAAA-3′	194	58	JN688205.1
*IGF-I*	F5′-ATCAGCAGTCTTCCAACCCA-3′ R5′-AGAGCATCCACCAACTCAGC-3′	179	58	NM_001285697.1
*DGAT1*	F5′-ACTACTACGTGCTCAACCGC-3′ R5′-AGACTGCAATCGCGTGTCG-3′	126	60	MT221183.1
*IL-6*	F5′-TTCAGTCCACTCGCTGTCTC-3′ R5′-TGCTTGGGGTGGTGTCATTC-3′	106	58	NM_001285640.1
*IL-8*	F5′-CTGGCCAGGATTCACGAGTT-3′ R5′-TGCTTCCACATGTCCTCACA-3′	117	60	XM_005681749.3
*TNF-α*	F5′-GCATGAGCACCAAAAGCATGA-3′ R5′-CTGGGGACTGCTCTTCCCTCT-3′	198	60	NM_001286442.1
*SOD1*	F5′-ATCCACTTCGAGGCAAAGGG-3′ R5′-CTGCACTGGTACAGCCTTGT-3′	124	60	NM_001285550.1
*CAT*	F5′-ACACAGGCACATGAACGGAT-3′ R5′-CCGTAGTCAGGGTCTTCGTG-3′	159	58	GQ204786.1
*GPx1*	F5′-AAGTTCATCACGTGGTCCCC-3′ R5′-CTGGGACAGCAGGGTTTCAA-3′	153	58	XM_005695962.3
*Nrf2*	F5′-CTACGGGCAAAAGCTCTCCA-3′ R5′-TCTGCAATTCTGAGCAGCCA-3′	171	60	KM576769.1
*β. actin*	F5′-CGTGCTGCTGACGGAGGCCCC-3′ R5′-GCACAGCCTGGATGGCCACATAC-3′	113	60	AF481159.1

**Table 4:** Effects of experimental blend of phytochemicals and lactic acid bacteria feed additive (PEL) on feed intake, nutrient digestibility, feeding value, and ruminal fermentation parameters of pregnant Zaraibi does.

	Control	PEL		SEM	*p*-Values	
	0 g/Day	2 g/Day	4 g/Day		Linear	Quadratic
Feed intake, g/day						
Dry matter	1089	1095	1104	1.02	<0.001	0.322
Total digestible nutrient	700	722	755	0.62	<0.001	0.145
Digestible crude protein	11.3	11.7	12.2	0.01	<0.001	0.258
Nutrient digestibility, %						
Dry matter	63.8	66.6	68.7	0.19	<0.001	0.006
Organic matter	64.2	67.0	69.1	0.18	<0.001	<0.001
Crude protein	66.1	68.3	70.6	0.22	<0.001	<0.001
Crude fiber	53.7	55.2	57.5	0.17	<0.001	0.827
Ether extract	60.2	63.5	68.4	0.19	<0.001	0.026
Nitrogen-free extract	67.9	69.1	71.3	0.19	<0.001	0.557
Feeding value, %						
Total digestible nutrients	58.6	60.0	62.2	0.12	<0.001	0.338
Digestible crude protein	7.89	8.15	8.43	0.03	<0.001	<0.001
Ruminal parameters						
pH values	6.43	6.35	6.31	0.030	0.003	0.699
Ammoina, mg/100 mL	21.5	20.5	19.7	0.10	<0.001	0.235
Volatial fatty acids (mmol/L)						
Acetic	46.8	49.8	52.1	0.12	<0.001	<0.001
Propionic	24.8	26.5	28.6	0.16	<0.001	0.388
Butyric	22.4	19.3	18.5	0.15	<0.001	0.001

SEM, standard error of mean.

**Table 5:** Effect of experimental blend of phytochemicals and lactic acid bacteria feed additive (PEL) on blood hematological parameters of Zaraibi does.

	Control	PEL		SEM	*p*-Value	
	0 g/Day	2 g/Day	4 g/Day		Linear	Quadratic
Hemoglobin, g/dL	7.30	8.86	9.67	0.110	<0.001	0.970
Red blood cells, ×10^6^/µL	6.35	6.88	7.11	0.042	<0.001	0.005
Hematocrit, %	32.1	35.5	38.3	0.453	<0.001	0.185
Mean corpuscular hemoglobin, pg/cell	27.8	24.8	23.9	0.431	<0.001	0.661
Mean corpuscular volume, µm^3^	19.2	18.8	18.2	0.224	<0.001	0.044
Mean corpuscular hemoglobin concentration, %	58.9	56.2	53.1	0.254	<0.001	0.492
Platelet count	465	475	485	1.120	<0.001	0.362
White blood cells, ×10^3^/µL	11.3	8.84	7.86	0.310	<0.001	<0.001
Neutrophils, %	42.2	50.6	55.7	0.540	<0.001	0.648
Lymphocytes, %	48.6	43.0	39.1	0.411	<0.001	0.696
Monocytes, %	4.71	3.86	2.86	0.262	<0.001	0.919
Eosinophils, %	2.73	1.63	1.31	0.181	<0.001	0.616

SEM, standard error of mean.

**Table 6:** Effect of experimental blend of phytochemicals and lactic acid bacteria feed additive (PEL) on blood serum parameters of Zaraibi does.

	Control	PEL		SEM	*p*-Value	
	0 g/Day	2 g/Day	4 g/Day		Linear	Quadratic
Serum protein, g/dL						
Total protein	5.39	5.96	6.97	0.030	<0.001	0.018
Albumin	3.16	3.37	3.79	0.021	<0.001	0.003
Globulin	2.23	2.60	3.21	0.032	<0.001	0.167
Albumin/Globulin ratio	1.42	1.30	1.18	0.021	<0.001	0.385
Liver function, IU/L						
Aspartate aminotransferase	43.9	41.4	40.2	0.110	<0.001	0.027
Alanine aminotransferase	30.2	28.1	25.9	0.201	<0.001	<0.001
Kidney function, mg/dL						
Urea	37.8	33.7	24.2	0.550	<0.001	0.009
Creatinine	1.21	0.95	0.82	0.011	<0.001	0.004
Serum lipids, mg/dL						
Total Cholesterol	130	122	116	0.911	<0.001	0.020
Triglyceride	92.2	86.2	77.3	0.468	<0.001	0.032
High-density lipoprotein	41.7	48.4	63.6	0.780	<0.001	<0.001
Low-density lipoprotein	61.9	54.5	46.4	0.851	<0.001	0.089
Very low-density lipoprotein	18.4	17.2	15.5	0.090	<0.001	0.032
Glucose	113.1	98.1	88.4	0.900	<0.001	<0.001
Lipase, U/L	46.7	108	129	0.983	<0.001	<0.001
Amylase, U/L	78.7	83.4	87.9	0.621	<0.001	0.049
Cortisol, ug/dL	1.57	0.84	0.47	0.040	<0.001	0.014
Thyroid hormones, ng/ml						
Triiodothyronine (T3)	0.52	0.48	0.33	0.010	<0.001	0.187
Thyroxine (T4)	29.4	30.3	38.4	0.440	<0.001	0.002

SEM, standard error of mean.

**Table 7:** Effect of experimental blend of phytochemicals and lactic acid bacteria feed additive (PEL) on some blood serum immunity, antioxidant, and inflammation indicators in Zaraibi does.

	Control	PEL		SEM	*p*-Value	
	0 g/Day	2 g/Day	4 g/Day		Linear	Quadratic
Immune indicators						
Immunoglobulin G, mg/dL	31.4	45.8	50.3	0.430	<0.001	<0.001
Immunoglobulin M, mg/dL	50.7	61.3	65.3	0.333	<0.001	<0.001
Antioxidant indicators						
Total antioxidant capacity (TAC), mmol/L	0.87	1.18	1.62	0.030	<0.001	<0.001
Malondialdehyde (MDA), nmol/mL	14.5	12.4	9.61	0.171	<0.001	0.041
Catalase (CAT), U/g	464.1	538.9	616.3	4.560	<0.001	0.920
Glutathione peroxidase (GPx), U/I	1.13	1.89	2.20	0.021	<0.001	<0.001
Inflammation indicators, pg/mg						
Interleukin B (ILB)	881.1	673.6	408.3	14.72	<0.001	0.095
Interleukin6 (IL6)	386.3	234.1	175.1	4.501	<0.001	<0.001

SEM, standard error of mean.

## Data Availability

The data supporting the results reported here are available at a reasonable request from the corresponding author.
